# E2Fs co-participate in cadmium stress response through activation of MSHs during the cell cycle

**DOI:** 10.3389/fpls.2022.1068769

**Published:** 2022-11-29

**Authors:** Wen-Jie Zheng, Wang-Qing Li, Yan Peng, Ye Shao, Li Tang, Ci-Tao Liu, Dan Zhang, Lan-Jing Zhang, Ji-Huan Li, Wu-Zhong Luo, Zhi-Cheng Yuan, Bing-Ran Zhao, Bi-Gang Mao

**Affiliations:** ^1^ Longping Branch, College of Biology, Hunan University, Changsha, China; ^2^ State Key Laboratory of Hybrid Rice, Hunan Hybrid Rice Research Center, Changsha, China; ^3^ College of Agricultural, Hunan Agricultural University, Changsha, China

**Keywords:** cadmium, cell cycle, E2Fs, MSHs, DNA damage, RNA-seq

## Abstract

Cadmium is one of the most common heavy metal contaminants found in agricultural fields. MutSα, MutSβ, and MutSγ are three different MutS-associated protein heterodimer complexes consisting of MSH2/MSH6, MSH2/MSH3, and MSH2/MSH7, respectively. These complexes have different mismatch recognition properties and abilities to support MMR. However, changes in mismatch repair genes (*OsMSH2*, *OsMSH3*, *OsMSH6*, and *OsMSH7*) of the MutS system in rice, one of the most important food crops, under cadmium stress and their association with E2Fs, the key transcription factors affecting cell cycles, are poorly evaluated. In this study, we systematically categorized six rice E2Fs and confirmed that *OsMSHs* were the downstream target genes of E2F using dual-luciferase reporter assays. In addition, we constructed four *msh* mutant rice varieties (*msh2*, *msh*3, *msh6*, and *msh*7) using the CRISPR-Cas9 technology, exposed these mutant rice seedlings to different concentrations of cadmium (0, 2, and 4 mg/L) and observed changes in their phenotype and transcriptomic profiles using RNA-Seq and qRT-PCR. We found that the difference in plant height before and after cadmium stress was more significant in mutant rice seedlings than in wild-type rice seedlings. Transcriptomic profiling and qRT-PCR quantification showed that cadmium stress specifically mobilized cell cycle-related genes *ATR*, *CDKB2;1*, *MAD2*, *CycD5;2*, *CDKA;1*, and *OsRBR1*. Furthermore, we expressed OsE2Fs in yeasts and found that heterologous E2F expression in yeast strains regulated cadmium tolerance by regulating *MSHs* expression. Further exploration of the underlying mechanisms revealed that cadmium stress may activate the CDKA/CYCD complex, which phosphorylates RBR proteins to release E2F, to regulate downstream *MSHs* expression and subsequent DNA damage repairment, thereby enhancing the response to cadmium stress.

## Introduction

Cadmium (Cd) is toxic to animals and plants. Extensive Cd contamination in soils can lead to cadmium accumulation in edible parts of crops, especially rice, threatening the food safety of rice consumption (Uraguchi and Fujiwara, 2012). To minimize the risk of soil Cd entering the food chain, understanding plants’ molecular response network to Cd and in-depth exploring the molecular mechanism of Cd stress have become an important direction in agriculture and environment studies (Pena et al., 2012; Wang et al., 2016; Chang et al., 2019).

The development of multicellular organisms relies on the orchestrated spatiotemporal regulation of cell division, including mitotic cell cycle and cell expansion. Therefore, cell cycles must be integrated into a complex histogenesis and organogenesis system (Mironov et al., 1997; Meijer and Murray, 2001; Stals and Inzé, 2001; Veylder et al., 2003). The plant cell cycle consists of four distinct phases: postmitotic interphase G1, DNA synthesis phase S, pre-mitotic interphase G2, and mitosis/cytokinesis phase M (Qi and Zhang, 2019). During the G1/S transition, the A-type cyclin-dependent kinase/Cyclin D (CDKA/CYCD) complex, *via* phosphorylating retinoblastoma-related (RBR) proteins, activates the S-phase E2 promoter binding factor (E2F). Activated E2F then regulates the expression of genes involved in DNA replication, cell cycle progression, and chromatin dynamics transition, thereby promoting the G1/S transition (del Pozo et al., 2006).

Both typical and atypical E2F transcription factors are key components of the cyclin D/RB/E2F pathway. Typical E2F proteins possess a homologous DNA-binding domain and form heterodimers with Dimerization part (DP) proteins to bind DNA *via* the leucine zipper in their dimerization region. In contrast, atypical E2F/DEL factors do not interact with DPs due to lacking a dimerization region. However, they have a homologous DNA-binding domain for replication, enabling them to bind DNA autonomously (Perrotta et al., 2021). In addition, typical E2F proteins also possess a conserved C-terminal transactivation domain that is absent in DPs and in atypical E2F/DEL proteins, allowing transcriptional activation of their target genes (Dimova and Dyson, 2005). *Arabidopsis* E2F/DP family includes three typical E2Fs (*AtE2Fa*, *AtE2Fb*, and *AtE2Fc*), three atypical E2Fs (*AtE2Fd/DEL2*, *AtE2Fe*/*DEL1*, and *AtE2Ff*/*DEL3*) and two DPs (Mariconti et al., 2002). Carrot E2F/DP family consists of four typical E2Fs, three atypical E2Fs, and three DPs (Perrotta et al., 2021). Yeast, which is widely used as an *in vivo* model to explore cell cycle response mechanisms, also utilizes E2F homologs MCB-binding factor (MBF) and SCB-binding factor (SBF) proteins to regulate G1/S transition and cell proliferation (Morshed et al., 2020). Previous studies have identified four E2Fs, three DPs, and two DP-E2F-like (DELs) in the rice genome, but little is known about their taxonomy and functions (Guo et al., 2007).

Proper cell division and cycling requires tight regulation of key cell cycle-related genes. RBR proteins act as negative regulators of E2F transcription factors and are crucial for E2F to function properly (Weintraub et al., 1992). The CDKA/CYCD complex regulates E2F/DP family activity *via* RBR proteins, which mediate G1/S transition. CDKA forms complexes with A, B, or D-type cyclins to drive G2/M transition (Gutierrez, 2009). The dramatic repression of Mitotic arrest deficient protein 2 (*MAD2*) expression may mediate G2/M arrest through a dual mechanism to regulate chromosome segregation (Cao et al., 2018). ATM and Rad3-related (*ATR*)is a key component of the G2/M checkpoint in plant cells and is activated by the mitogen-activated protein kinase (MAPK) signaling pathway *via* phosphorylation (Yoshioka et al., 2006).

Many replication- and mismatch repair-related genes in *Arabidopsis* contain conserved E2F binding sequences in their predicted promoter regions (Kosugi and Ohashi (2002); Vandepoele et al., 2005). Previous work on the *Arabidopsis* genome at E2F binding sites has identified over 180 potential E2F target genes associated with transcription, stress, DNA damage, plant defense, and signaling transduction, in addition to the cell cycle (Ramirez-Parra, 2003). Among them are genes related to mismatch repair (MMR), such as Mutated S homologue 3 (*MSH3*), Mutated S homologue 6 (*MSH6*), and Postmeiotic segregation increased 2 (*PMS2*). In addition, the putative rice homologs are listed.

The most important role of the MMR system is to identify and correct mispaired or unpaired bases (Schofield and Hsieh, 2003; Iyer et al., 2006). Eukaryotic cells rely on high-fidelity DNA replication to maintain genomic integrity and depend on DNA MMR to ensure proofreading of incorrect pairings. Thus, any deletion in MMR genes can lead to spontaneous mutations in organisms (Zhang et al., 2005). In addition to correcting base-base mismatches, MMR genes are involved in suppressing mutations and inducing protective responses to various types of DNA damage. MMR plays multiple roles in response to various DNA damage inducers, such as nucleotide methylation, oxidative DNA damage, and UV-induced DNA damage, among other degenerative damages (Ijsselsteijn et al., 2020). Interestingly, cadmium interacts with DNA repair systems, cell cycle checkpoints, apoptosis-related epigenetic factors, and factors controlling gene expression. Cadmium can bind directly to DNA at very low concentrations, inducing various DNA damages such as base-base mismatches, insertion/deletion loops, strand cross-links, and breaks (Filipic, 2012). DNAs under stress can induce both complex and precise repair mechanisms and signal transduction pathways in eukaryotic cells and a stage-specific arrest of the cell cycle process (Wang et al., 2013; Hu et al., 2016; Xiang et al., 2017). Mutated S α (MutSα), Mutated S β (MutSβ), and Mutated S γ (MutSγ) are three different MutS-associated protein heterodimer complexes consisting of MSH2/MSH6, MSH2/MSH3, and MSH2/MSH7, respectively. These complexes have different mismatch recognition properties and abilities to support MMR (Wang et al., 2020). In plants, Knockdown of OsMSH2 does not cause gene variants at other locations in the genome (Karthika et al., 2021). Mutated S homologue 2 (MSH2) preferentially activates ATR to trigger DNA damage responses (DDR), including regulation of cell cycle, endoreplication, cell death, and recruitment of other DNA repair, thereby enhancing plant tolerance to cadmium (Wang et al., 2020). OsMSH6 affects rice microsatellite stability and homologous recombination and plays an important role in ensuring genome stability and genetic (Jiang et al., 2020). MSH7 is a plant-specific protein similar to MSH6, and in rice OsMSH7 is able to interact with Meiotic chromosome association 1 (MEICA1) and play a role in meiotic recombination (Hu et al., 2017). *AtMSH2* and *AtMSH6* are involved in G2/M or G1/S transitions in *Arabidopsis* and soybeans. *MSH2* and *MSH6* may be the direct sensors of cadmium-mediated DNA damage. Expression of DNA mismatch repair-related genes AtMSH2, AtMSH3, AtMSH6, and AtMSH7 can be used as potential biomarkers for evaluating cadmium exposure in Arabidopsis seedlings (Liu et al., 2009).

In this study, we systematically classified E2Fs in rice, determined its relationship with downstream MSH target genes, and then explored the phenotype of rice E2F and MSH under cadmium stress. These conclusions combined with transcriptome and quantitative results, established a model hypothesis of E2Fs-MSHs.The study revealed that E2Fs co-participate in responses to cadmium stress by binding to MSHs during the cell cycle.

## Materials and methods

### Plant materials and growth conditions

Indica rice variety Huazhan (HZ) grains were obtained from the State Key Laboratory of Hybrid Rice. *Osmsh2*, *Osmsh3*, *Osmsh6*, and *Osmsh7* knockout rice were established using the CRISPR-Cas9 technology *via* transformation, screened by hygromycin, and sequencing verified in each generation. The pure T3 generation seeds were obtained and used for subsequent experiments. All plant materials were grown in the transgenic test base, and seedlings were cultured in an artificial climate chamber (Hunan Changsha).

The *Osmsh2* exon 4, *Osmsh3* exon 2, *Osmsh6* exon 1, and *Osmsh7* exon 2 were selected as the target sites to construct the gene knockout vectors pYLCRISPR/Cas9-MT(I)-*OsMSH2*, *OsMSH3*, *OsMSH6*, and *OsMSH7*, respectively, using target connector primers Cas9-*OsMSH2*-F and Cas9-*OsMSH2*-R and the target-linked primers Cas9-F and Cas9-R. All primer pairs are listed in **Supplemental Table 1**. Accession numbers for all genes are listed in **Supplementary Table 2**.

Rice seeds were germinated at 37°C and 60% relative humidity after sterilization first in H_2_O_2_ (10% v/v) for 0.5 h and then in NaClO (0.1% v/v) for 1 day and transferred to 96-well nursery plates. Seedlings were cultivated in 1/4 nutrient solution as recommended by the International Rice Research Institute (Ponnamperuma, 1977). The 10-day-old seedlings were subjected to CdCl_2_ treatment at 0, 2, and 4 mg/L concentrations for 10 days. The seedling leaves were collected at 6 hours of treatment, snap-frozen in liquid nitrogen, and stored at -80°C for RNA extraction. Total RNAs were extracted from seedling leaves using an RNAprep pure Plant Kit (Magen, China) and reverse transcribed using a SuperScript II kit (TransGen, China). RNA concentration and quality were determined with a NanoDrop 2000 (Thermo Scientific, Waltham, MA, USA). Each RNA sample was divided into two aliquots for RNA-seq and qRT-PCR, respectively.

### Phylogenetic tree construction, gene structure, protein structure and expression pattern analysis

Clustal_W was used to compare the E2F sequences of Arabidopsis Thaliana and rice, and GeneDoc was used to output the amino acid alignment map. The phylogenetic tree was constructed using the adjacency method (NJ) of MEGA6.0 software, and the Bootstrap value was set to 1000. From the Ensembl the Plants get the length of the candidate genes or cDNA sequence, and CDS use GSDS 2.0 (http://gsds.cbi.pku.edu.cn/) to analyze E2F gene exon/embedded substructure, The conserved domain was analyzed using MEME tool (http://meme-suite.org/index.html). From the Rice Expression database IC4R (Information Commons for Rice; The expression levels of E2F genes in different tissues were obtained in http://ic4r.org, and then plotted with TBtools (Chen et al., 2020).

### Dual-luciferase reporter assays

The full-length coding regions of *OsE2Fa-1*, *OsE2Fa-2*, *OsE2Fa-3*, *OsE2Fc*, *OsE2Fe-1*, and *OsE2Fe-2* were amplified using specific primers listed in **Supplemental Table 1** and cloned into a pGreen II 62-SK vector. After that, pGreenII 0800-LUC double-reporter vector was fused to the promoter fragments of *OsMSHs*. The above constructs were transferred into *A. tumefaciens* strain GV3101 (pSoup-p19) to generate pro35S:*OsE2Fa-1*, pro35S:*OsE2Fa-2*, pro35S:*OsE2Fa-3*, pro35S:*OsE2Fc*, pro35S:*OsE2Fe-1*, pro35S:*OsE2Fe-2*, proOsMSH2:LUC, proOsMSH3:LUC, proOsMSH6:LUC, and proOsMSH7:LUC recombinant strains. *N. benthamiana* leaves were co-infiltrated with pro35S:E2F and proOsMSH2/3/6/7:LUC and cultivated for 3 days in a growth chamber. *N. benthamiana* leaves infiltrated with proOsMSH2/3/6/7:LUC were used as the internal control. After inoculation and a transient incubation of 72 h, the relative LUC activity was measured using a dual-LUC reporter assay system (Promega), which included firefly LUC and Renilla (REN) LUC. Leaf discs with an area of 2 cm^2^ leaf were sampled and finely ground in 500 mL of Passive Lysis Buffer. Crude extracts (8 μL) were mixed with 40 μL of Luciferase Assay Buffer (Hellens et al., 2005), and the promoter activity was determined as the LUC/REN value using a luminometer (Modulus™, Promega). Each measurement included three independent biological replicates.

### Heterologous OsE2Fs expression in yeasts

The full-length ORFs of OsE2Fs were amplified from *Oryza sativa* Nipponbare cDNA using primer pairs listed in **Supplemental Table 1**, digested with *Kpn*I and *Bam*HI and cloned into the corresponding sites of yeast expression vector pYES2 (Invitrogen). The resulting pYES2-OsMSHs constructs and the empty vector were transformed into yeast strain Δycf1 (BY4741; *MATa*; *his3Δ1*; *leu2Δ0*; *met15Δ0*; *ura3Δ0*; *YDR135c::kanMX4*) (Tan et al., 2019). Positive OsE2F transformants were selected on synthetic media lacking uracil. To analyze cadmium tolerance, individual transformant cultures were diluted, spotted on solid media containing 2% galactose and 0, 5, 10, or 20 μM CdCl_2_, incubated at 30°C for 4 days, and photographed.

### Subcellular localization

Subcellular localization of OsMSHs was investigated by transiently overexpressing 35S:OsMSH2-GFP, 35S:OsMSH3-GFP, 35S:OsMSH6-GFP, and 35S:OsMSH7-GFP in tobacco (Nicotiana tabacum) leaves *via* Agrobacterium-mediated transformation (Sparkes et al., 2006). Nuclear marker (m-Cherry) and GFP signals were observed under a confocal scanning microscope (Model LSM 880, Zeiss, Jena, Germany).

### Transcriptome sequencing analysis

#### RNA quality control, illumina library construction, and sequencing

mRNA samples were isolated using VAHTSTM mRNA Capture Beads (Vazyme Biotech) and analyzed using Qubit (Invitrogen) and bioanalyzer (Shanghai Furi Science and Technology) to determine their concentration and contamination. After that, RNA-sequencing libraries were established. Briefly, the 1^st^ strand cDNAs were synthesized using the 1^st^ strand buffer and enzyme mix, and the 2^nd^ strand DNAs were subsequently obtained by adding the 2^nd^ strand buffer and enzyme mix. Double-stranded DNAs were purified using 1.8×VAHTS™ DNA Clean Beads (Vazyme Biotech) and subjected to terminal repair and elongation with dA-tailing, as well as ligation with adaptors. The target fragments were size-selected by 0.7× and 0.1×VAHTS™ DNA Clean Beads (Vazyme Biotech), amplified as sequencing templates and sequenced on an Illumina HiSeq™ system (LC Sciences) following the manufacturer’s protocol. Clean reads were mapped to a reference genome (*Oryza sativa* Group 4.0, https://www.ncbi.nlm.nih.gov/nuccore/255672756?report=fasta), and gene expression levels were calculated by quantifying the cDNA fragments per kilobase of transcript per million fragments mapped (FPKM).

#### Quantitative real-time reverse transcription-PCR

Quantitative real-time reverse transcription-PCR (qRT-PCR) was performed in a 384-well plate using the SYBR premix Ex TaqTM kit (Vazyme, China) on a Roche LightCycler 480 II instrument. Relative gene expression levels were analyzed using the 2^-ΔΔCt^ method (Livak and Schmittgen, 2001).

#### Differentially expressed genes and heatmap expression of FPKM of related genes

Differentially expressed genes (DEGs) were identified using the DESeq method based on negative binomial distribution with an absolute log2 (fold change) value ≥ 1 and the corresponding Q value ≤ 0.05 as the selecting thresholds. The Q value was a corrected p-value calculated using KOBAS 2.0 and the BH method (Mao et al., 2005). The FPKM values of the screened related genes were summarized as heatmap expression using TBtools (Chen et al., 2020).

#### Data analysis

Statistical analysis was performed using SPSS (version 20.0). Differential expression analysis was performed for each group of samples using the DESeq2 R package (1.20.0). One-way analysis of variance and the least significant difference test were used to detect significant differences between mutant and wild-type rice.

## Results

### Evolutionary analysis and tissue-specific expression profiles of the E2F family in *Arabidopsis* and rice

**Figure 1:**
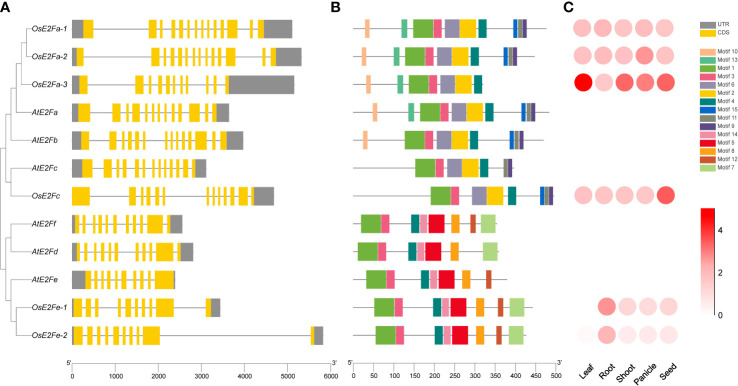
Phylogenetic tree, gene structure, conserved structural domains, and expression pattern analysis of rice and *Arabidopsis* E2Fs **(A)** On the left is the phylogenetic tree of the rice and *Arabidopsis* gene families. The gene structures are shown on the right. Exons and UTRs are indicated by boxes, while gray lines indicate introns of genes, respectively. **(B)** The distribution of motifs in the amino acid sequence. Patterns are indicated by different colored boxes. The scale at the bottom indicates the gene length. **(C)** Expression patterns of rice E2Fs in different tissues, with white indicating the lowest expression and red the highest.

Six rice E2F genes were identified by screening and E2F candidate genes in the *Arabidopsis* and rice E2F transcription factor database using *Arabidopsis* and rice Pfam (PF02319) (Mariconti et al., 2002; Guo et al., 2007; Perrotta et al., 2021) and renamed according to the *Arabidopsis* nomenclature (**Figure 1A**). Their exon structure is similarly complex to that of *Arabidopsis*, with the least number of *OsE2Fe-1* exons, 10. MEME(Motif-based sequence analysis tools) analysis of E2F proteins identified 15 conserved motifs, and their distributions in rice and *Arabidopsis* are shown in **Figure 1B**. Conservative motif analysis showed that most motifs clustered in the same phylogenetic taxon share a common motif composition, suggesting that most evolutionarily-related motifs may have the same physiological functions. The similarity of gene structures and motif compositions verifies that OsE2F branching and classification in this study are reliable. The expression patterns of each rice E2F gene in leaves, roots, stems, panicles and seeds were downloaded from the Rice Expression Database (http://expression.ic4r.org/) (Project ID: DRP000391) and presented as a heatmap (**Figure 1C**). It is obviously that the expression of *OsE2Fe-1* and *OsE2Fe-2* was extremely lower in rice leaves but very similar among other tissues. The amino acid sequence comparison revealed no significant difference in DNA binding-1, DNA binding-2, pRBR binding, leucine zipper, and marked box between *Arabidopsis* and rice E2F genes. Our findings (**Figure S1**) complement and refine the previous work, which only compared individual rice E2F genes Kosugi and Ohashi (2002).

### E2F transcription factor can directly or indirectly activate MSH genes

**Figure 2:**
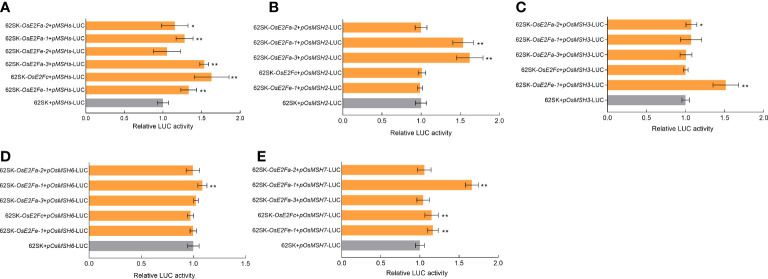
**(A)** LUC activation of OsE2Fs and MSHs. **(B-E)** LUC activation of OsE2Fs with OsMSH2,OsMSH3,OsMSH6 and OsMSH7, respectively. Transient dual luciferase reporter analysis indicates that E2Fs can activate MSHs. 62SK represents empty pGreenII 62-SK vector. 62SK-OsE2F represents the pGreenII 62-SK-OsE2F vector. pMSH-LUC represents pGreenII 0800-pMSH-LUC vector. Renilla luciferase (REN) was used for normalization. Results shown are means G SD (n = 9). Asterisks show significant differences from the control (Student’s t-test, p < 0.05). * indicates p<0.05, ** indicates p<0.01.

To verify the activation status of MSHs gene by E2F transcription factor, possible transcription factor binding sites in the promoter regions of *OsMSH2*, *OsMSH3*, *OsMSH6*, and *OsMSH7* were analyzed and predicted using Plant Care. All binding sites were formed into a tandem sequence as the target promoter and constructed into LUC vectors to examine their binding with E2Fs in 62SK (Hellens et al., 2005). The results showed that all E2Fs, except OsE2Fe-2, could directly or indirectly activate the target genes (**Figure 2A**). The tandem element was then split into *OsMSH2* (TTTGCCGCTTTCCCGC), *OsMSH3* (TTTCCCGC), *OsMSH6* (TTTCCCGC), and *OsMSH7* (GCGGGAAATTTCCCGC), and their binding with each E2F was determined separately. The results showed that OsE2Fa-1 and OsE2Fa-3 could directly or indirectly activate *OsMSH2* (**Figure 2B**), OsE2Fa-2 and OsE2Fe-1 can activate *OsMSH3* directly or indirectly (**Figure 2C**), OsE2Fa-1 activates *OsMSH6* (**Figure 2D**), and OsE2Fa-1, OsE2Fc and OsE2Fe-1 activates *OsMSH7* (**Figure 2E**). Taken together, most E2F transcription factors activate MSH7, while OsE2Fa-1 activates most MSHs. These results are consistent with the previous prediction for the E2F binding sites (Vandepoele et al., 2005).

### MSH genes are involved in cadmium stress response

**Figure 3:**
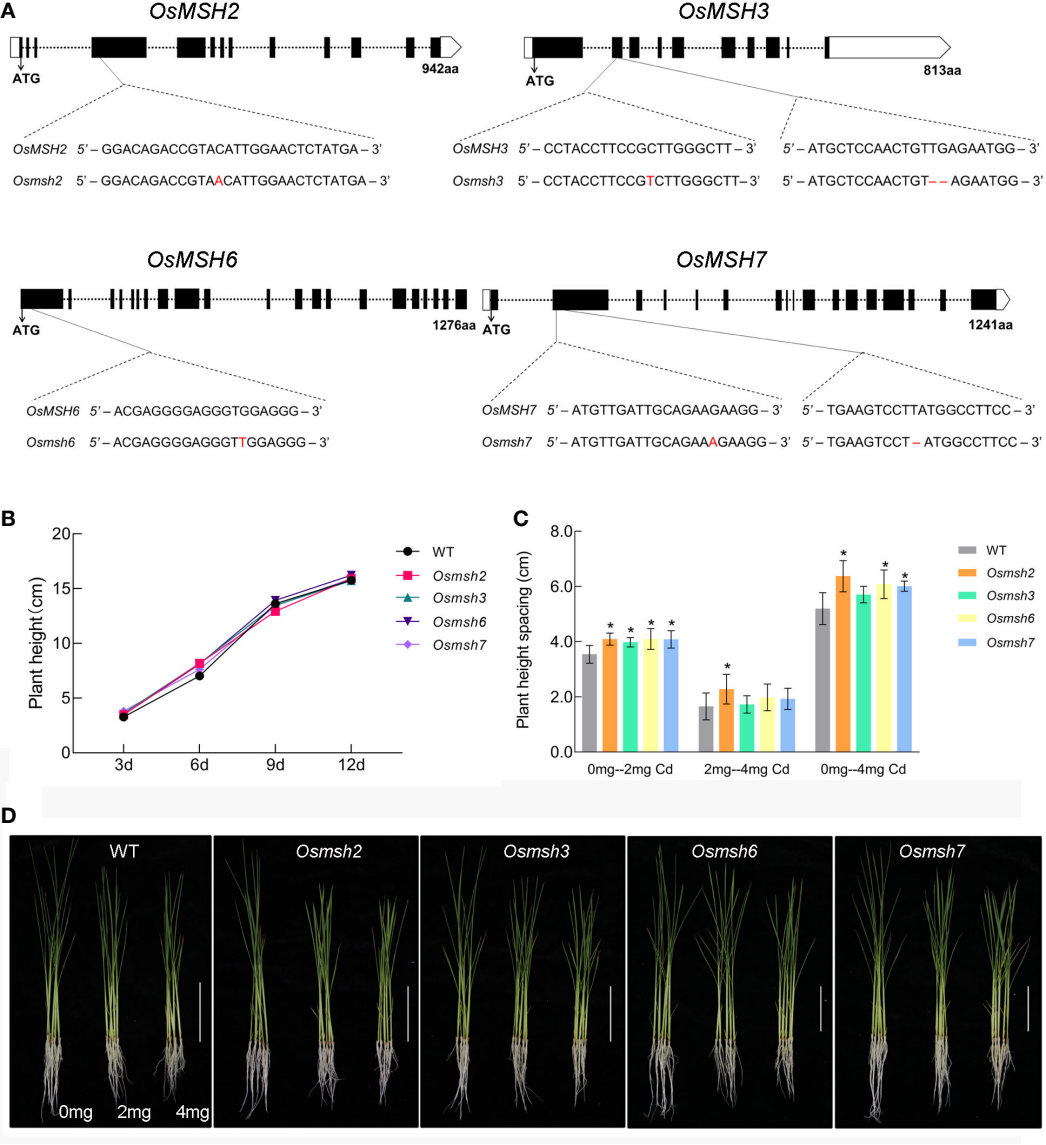
Phenotype of rice mutant seedlings under cadmium stress. **(A)** Gene structure of the rice MSH gene and editing loci for mutants. **(B)** Growth curves of rice msh mutant and wild-type seedlings from the start of germination to the 12-day stage. **(C)** Statistics of plant height differences between rice msh mutant and wild-type seedlings under 2 mg and 4 mg stress. (Student’s t-test, p < 0.05). **(D)** Phenotypic photographs of rice msh mutant and wild-type seedlings under 2 mg and 4 mg stress(n = 5 Scale bars: 10 cm). * indicates p<0.05.

Rice mutant materials *Osmsh2*, *Osmsh3*, *Osmsh6*, and *Osmsh7* were created using the CRISPR-Cas9 system. **Figure 3A** shows the gene structures and editing types. To ensure the accuracy of the experiment and the consistency of variables, the height of wild-type and mutant rice seedlings without cadmium treatment were measured at the cycle point of rice growth till day 12 and showed no significant difference (**Figure 3B**). We also calculated the germination percentage of wild-type and mutant seeds before treatment. Treatment with 2 mg/L or 4 mg/L CdCl_2_ significantly reduced the height, root length, fresh weight, and dry weight of wild-type and mutant rice seedlings (**Supplemental Table 3** **)**. After 10 days of 2 mg/L CdCl_2_ treatment, but not after 10 days of 4 mg/L CdCl_2_ treatment, the height of rice seedlings was significantly different between wild-type seedlings and *Osmsh2*, *Osmsh3*, *Osmsh6*, and *Osmsh7* mutants. Moreover, the height of *Osmsh2* mutant seedlings, but not that of wild-type and other mutant seedlings, was significantly different after 2 mg/L and 4 mg/L CdCl_2_ treatment (**Figure 3C**). Furthermore, the height of all mutant seedlings except *Osmsh3* without CdCl_2_ treatment was significantly different from that after 4 mg/L CdCl_2_ treatment (**Figure 3D**). There were no statistically significant changes in root length, fresh weight, and dry weight between wild-type and mutant seedlings after 2 mg/L and 4 mg/L CdCl_2_ treatment (**Figure S2D**). These results indicate that *OsMSH2*, *OsMSH3*, *OsMSH6*, and *OsMSH7* are involved in cadmium stress response in rice.

### MSHs affect cell cycle progression under cadmium stress

**Figure 4:**
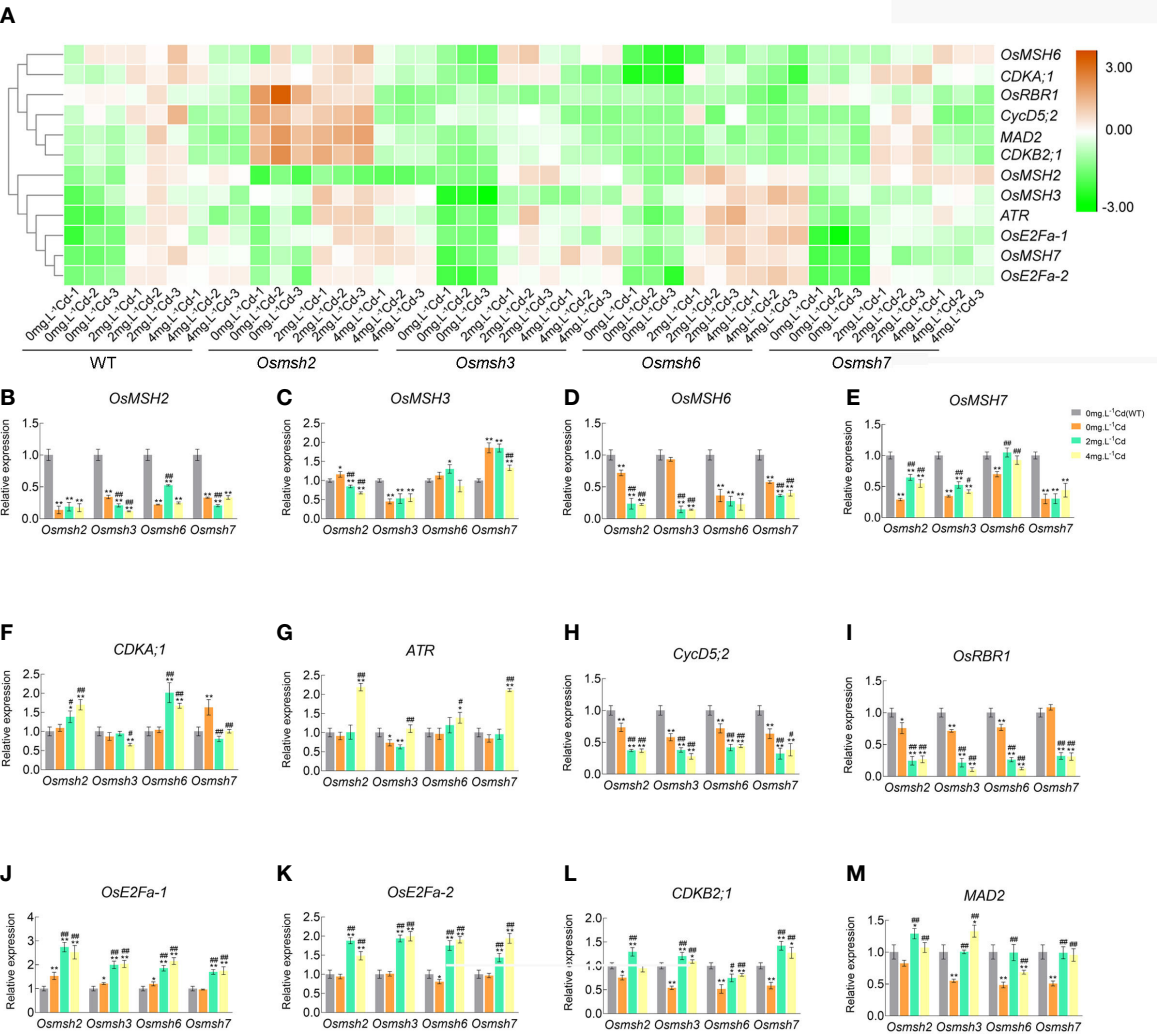
Differential expression profiles and quantitative validation of cell cycle-related genes in response to Cd stress in rice *Osmsh2*, *Osmsh3*, *Osmsh6* and *Osmsh7* mutant leaves. **(A)** Heat map representing some of the 12 cell cycle-related DEGs (P-adj≤ 0.05 and log2-fold change ≥ 1.5). Each row shows the relative expression levels of individual genes and each column shows the expression levels of individual samples. **(B–M)** The expression levels of the WT were set to 100% in the control by qRT-PCR analysis. Data were shown mean ± SD at least three independent experiments, and house-keeping gene Osactin was used as an internal control. * and ^#^ significantly statistical difference from the WT control and the corresponding mutant control, respectively (P < 0.05). ** and ## were significantly different from WT control and WT mutant control (P < 0.01).

The effects of MSH genes on cell cycle progression under cadmium stress in rice leaves were determined by RNA-seq analysis to identify cell cycle genes with differential changes and further verified by qRT-PCR. The results showed that the expression of all MSH genes was upregulated in wild-type rice leaves after cadmium treatment. The expression of cell cycle-related genes (*CDKB2;1*, *ATR*, *MAD2*, *OsE2Fa-1*, *OsE2Fa-2*, *CycD5;2*, and *CDKA;1*) in rice leaves under increasing cadmium stress showed an inverted “U” pattern, with a 1.0-2.5fold upregulation at 2 mg/L CdCl_2_ and a 0.8-1.9fold upregulation at 4 mg/L CdCl_2_. The expression of *OsRBR1* was upregulated by 1.5-2.5 and 0.8-1.9 folds at 2 mg/L and 4 mg/L CdCl_2_, respectively. Unlike other genes, *OsRBR1* showed a significant downregulation at 4 mg/L CdCl_2_ and no significant difference at 2 mg/L CdCl_2_. It is worth noting that *OsMSH2*, *OsMSH3*, *OsMSH6*, and *OsMSH7* showed a stepwise increase in expression at 2 mg/L and 4 mg/L CdCl_2_. The expression of these genes was upregulated by 1.9-2.6 folds under 2 mg/L CdCl_2_ and 2.2-3.3 folds under 4 mg/L CdCl_2_, indicating that plants would activate the MMR system to avoid more serious damages when exposed to toxic cadmium (**Figures S2A-C**). Cadmium stress strongly activated the expression of *ATR*, *CDKB2;1*, *MAD2*, *OsE2Fa-1*, and *OsE2Fa-2* in rice leaves deficient in *OsMSH2*, *OsMSH3*, *OsMSH6*, or *OsMSH7* compared with controls, whereas the expression of *CycD5;2* and *OsRBR1* was dramatically reduced in leaves of cadmium-stressed rice (**Figure 4**). Expression of *CDKA;1* was activated in leaves of *Osmsh2* and *Osmsh6* mutant rice.

The expression of *OsMSH3* and *OsMSH6* was significantly downregulated while *OsMSH7* was upregulated in *Osmsh2* mutant leaves under cadmium stress; In *Osmsh3* mutant leaves, the expression of *OsMSH2* and *OsMSH6* was significantly downregulated and *OsMSH7* was similarly activated; in *Osmsh6* mutant, *OsMSH2* and *OsMSH7* expression was upregulated to some extent; in *Osmsh7* mutant, *OsMSH2* and *OsMSH6* expression was slightly suppressed. The results of qRT-PCR were consistent with the trend of transcriptome sequencing, where most genes related to the cell cycle in each mutant were repressed under cadmium stress. Interestingly, the genes mobilized by cadmium stress included the key component genes of G1/S and G2/M, suggesting that cadmium stress affects cell cycle in rice. Venn diagram plots of DEGs showed that *Osmsh2* mutant has the most downregulated genes under 4 mg/L CdCl_2_ treatment, 1.3 times more than the wild-type (**Figure S3A**), of which cell cycle**-**specific genes account for 31% of the total downregulated genes. *Osmsh7* mutant has the most upregulated genes under 2 mg/L CdCl_2_, 1.7 times more than the wild-type, of which cell cycle-specific genes account for 57% of the total upregulated genes. *Osmsh6* mutant has 1.3 times more cell cycle-specific upregulated genes under 4 mg/L CdCl_2_ than the wild-type and 1.3 times more cell cycle-specific upregulated genes than the wild-type under 4 mg/L CdCl_2_. The difference in number of DEGs between *Osmsh3* mutant and wild-type was insignificant (**Figure S3B**).

### E2F transcription factors can respond to cadmium stress

**Figure 5:**
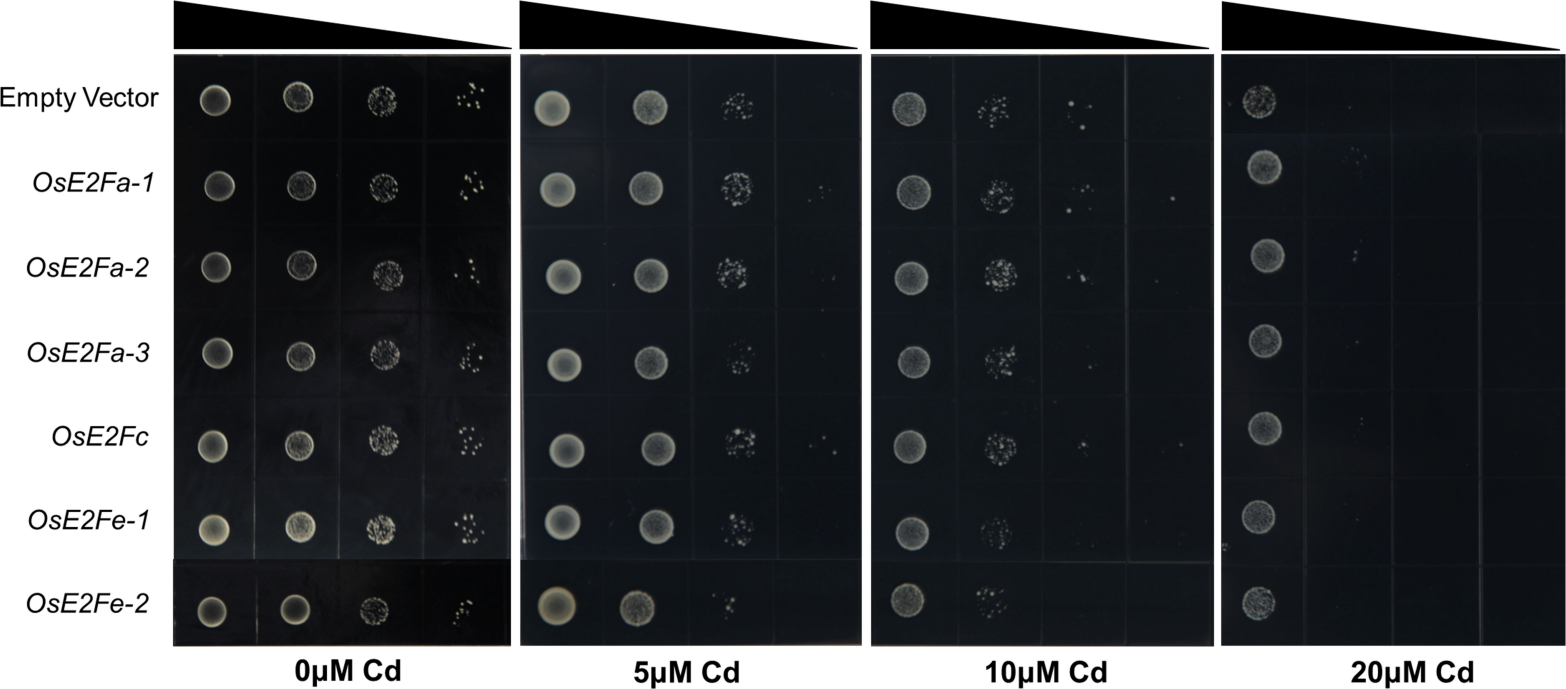
Expression of rice E2F members in Cd ion-sensitive mutant strain Δycf1. The empty vector, the control was pYES2, was grown in SD-Ura medium with the PYES2 vector linked with different E2F genes of rice. Induction of different levels of Cd stress was achieved using 0, 5, 10 and 20 μM of Cd.

Experiments with cadmium-sensitive yeast mutant strains showed that without cadmium, E2F expression did not affect the growth of yeast strains. However, under 5 μM cadmium stress, yeast strains expressing *OsE2Fa-1*, *OsE2Fa-2*, and *OsE2Fc* grew better than those expressing no or other E2F transcription factors. Similar results were observed under 10 μM cadmium stress, indicating that the yeast expression system is stable and the response trend of each E2F gene of Cd-sensitive strains to different cadmium stress is consistent. Understandably, this difference caused by gene expression was no longer significant at 20 μM cadmium stress (**Figure 5**).

### MSHs are localized in the nucleus

**Figure 6:**
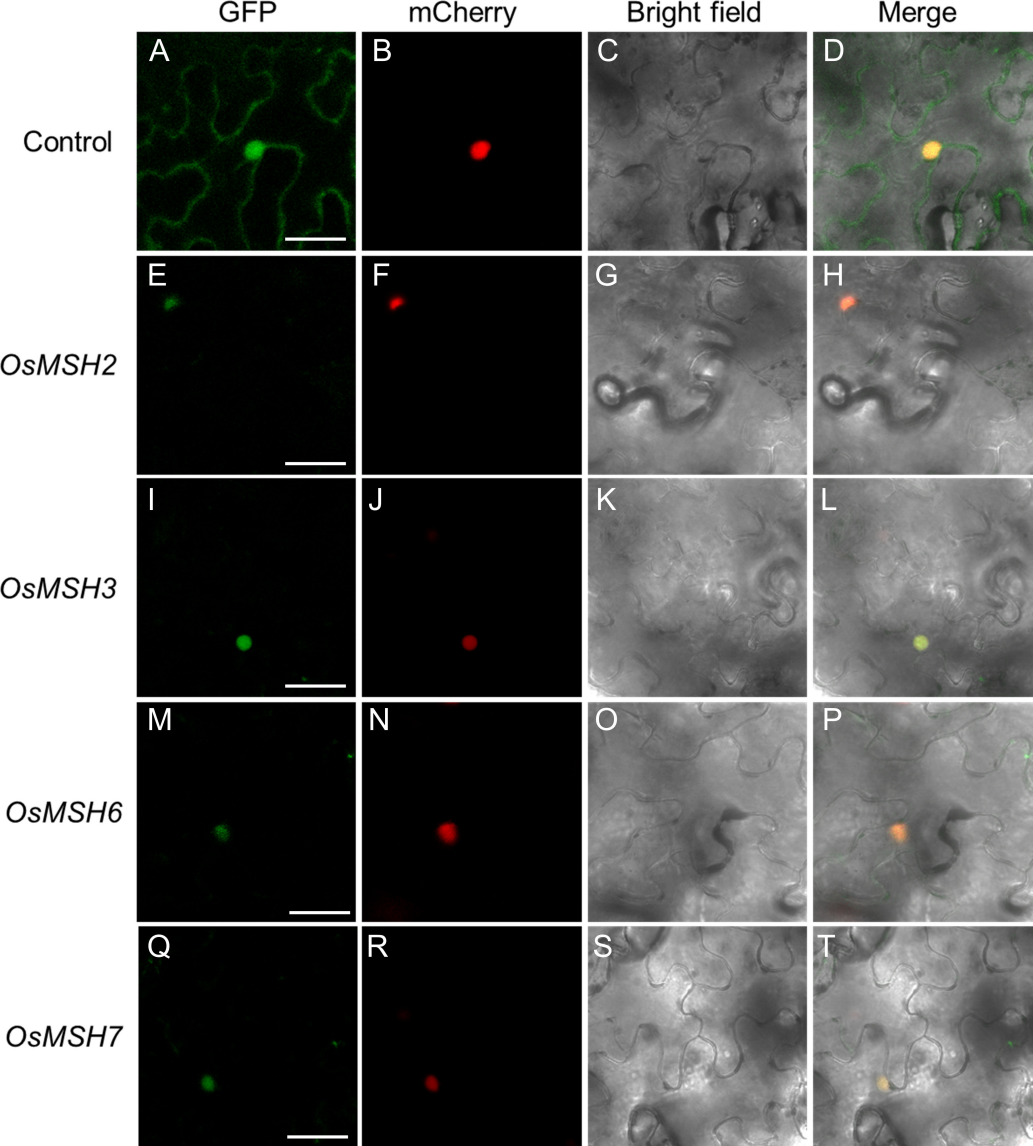
Subcellular localization of OsMSH2, OsMSH3, OsMSH6 and OsMSH7. **(A)** 35S::GFP control vector. **(E)** 35S::OsMSH2::GFP. **(I)** 35S:: OsMSH3::GFP. **(M)** 35S:: OsMSH6::GFP. **(Q)** 35S:: OsMSH7::GFP. **(B–R)** AtWRKY25-mCherry: a marker anchored in the cell nucleus. **(C–S)** Bright field. **(D–T)** Merged images. Scale bars: 20 μm.

To determine the subcellular localization of *OsMSH2*, *OsMSH3*, *OsMSH6*, and *OsMSH7*, *OsMSH2/3/6/7*-GFP fusion proteins driven by the CaMV 35S promoter were transiently co-expressed with the nuclear marker *AtWRKY25*-mCherry in tobacco. Confocal microscopic observation indicated that GFP signal co-existed with *AtWRKY25*-mCherry signals, indicated that *OsMSH2/3/6/7* proteins are localized in the nucleus (**Figure 6**).

## Discussion

The E2F transcription factor is a key component of the RB/E2F pathway, which is controlled by cyclin-dependent kinases and regulates cell-cycle progression in plants and animals (Perrotta et al., 2021). Plant homologues of E2F have been tentatively identified in rice and *Arabidopsis* (Kosugi and Ohashi (2002); Mariconti et al., 2002), In this report, we extend the information on E2F factor in rice and conclusively confirmed the previous prediction (Ramirez-Parra, 2003; Vandepoele et al., 2005) that E2Fs can target and bind to MSHs, MutS is a key link in the MMR system. Plants lacking MutS will bypass MMR-mediated DDR, thereby reducing the tolerance of plants to cadmium. This has been shown in *soybeans* and *Arabidopsis* (Cui et al., 2017; Cao et al., 2018; Zhao et al., 2020), our study further revealed that E2F improves rice’s tolerance to cadmium stress by binding to MSH, indicating that E2F together with MSHs responds to cadmium stress in the cell cycle. To the best of our knowledge, this is the first study on the relationship between E2F/MSH and cadmium stress in rice. The model developed here provides a preliminary explanation of the response mechanism of the E2F/MSHs pathway to cadmium stress in rice and a theoretical basis for creating cadmium-tolerant rice varieties.

Through the evolutionary tree analysis and amino acid sequence comparison of rice and *Arabidopsis* E2F transcription factors, we found that rice *OsE2Fa-1*, *OsE2Fa-2*, *OsE2Fa-3*, and *OsE2Fc* are functional transcription factors belonging to typical E2Fs (**Figure 1**), which, like *AtE2Fs*, have conserved C-terminal trans-activation domains and can specifically recognize E2F cis-elements and activate downstream target genes. By contrast, rice *OsE2Fe-1* and *OsE2Fe-2* are atypical E2Fs that possess DNA binding sites but lack transcriptional activation domains and cannot activate downstream gene expression. This further illustrates the complex regulatory mechanism of E2F transcription factors in rice cells (Rossi and Varotto, 2002).

Our LUC results showed that the tandem sequence consisting of cis-elements could bind to four typical transcription factors (*OsE2Fa-1*, *OsE2Fa-2*, *OsE2Fa-3*, and *OsE2Fc*) and atypical *OsE2Fe-1*, which also confirms that atypical E2F does not affect DNA binding in the absence of the transcriptional activation domain. Our activation assays showed that other rice E2F can directly or indirectly activate s *OsMSH2*, *OsMSH3*, *OsMSH6*, and *OsMSH7*, except *OsE2Fe-2*. These results confirmed the prediction that the cis-acting element of each gene could bind to 1-3 transcription factors and validated previous microarray analysis of the predicted target loci. In addition to the previously predicted *OsMSH3* and *OsMSH6*, our results suggest that *OsMSH2* and *OsMSH7* are also the targets of E2F (Vandepoele et al., 2005).

OsE2Fa-1 activates to OsMSH2, OsMSH6, and OsMSH7 (**Figure 2**), where OsMSH2 and OsMSH6 form MutSα heterodimers. MutSα recognizes single base mismatches such as polymerase errors, small insertion/deletion loops, and oxidative mismatches and methylation mismatches, which may be caused by translocation synthesis (TLS), (Zhang et al., 2002; Iyer et al., 2006; Campregher et al., 2008; Lippard, 2009). MSH2 and MSH7 form MutSγ heterodimers, which mainly recognize single base mismatches, including G/G, G/A, A/A, and C/A mismatches (Culligan and Hays, 2000; Wu et al., 2003; Gomez and Spampinato, 2013). In addition, MSH2 and MSH3 form MutSβ heterodimers, which sense insertion/deletion cycles and inter-strand cross-links (ICLs) (Marti et al., 2010). All of these suggest that *OsE2Fa-1* is a key transcription factor that activates MMR-related genes in canonical E2F. Other canonical E2F genes such as *OsE2Fa-2* activation of *OsMSH3* and *OsE2Fc* activation of *OsMSH7* also fully demonstrate that canonical E2F does not act alone and needs to cooperate with other MMR genes in MutSα, MutSβ, and MutSγ heterodimers.

The cadmium stress tests of the mutant and wild-type rice showed that the height of plants without cadmium stress and under 2 mg/L cadmium stress differed the most, possibly because rice leaves were most sensitive to 2 mg/L CdCl_2_. At CdCl_2_ > 2 mg/L, plants’ defense system against cadmium stress was damaged, leading to no significant difference (**Figure 3**). The differences in plant height between wild-type and mutant rice under different cadmium stresses reflected the important roles of *OsMSH2*, *OsMSH3*, *OsMSH6*, and *OsMSH7* in rice response to cadmium stress, indicating that MutS is involved in rice cadmium stress response. In *Arabidopsis*, the root growth of MSH2 or MSH6-deficient seedlings under Cd stress (1.25-4.0 mg/L) was much more inhibited than that of wild-type seedlings, which was consistent with our results (Cao et al., 2018).

Yeast heterologous expression assays of E2F genes showed significant differences in the growth of E2F-expressing yeast strains under 5 μM and 10 μM cadmium treatments, with yeast strains expressing *OsE2Fa-1*, *OsE2Fa-2* and *OsE2Fc* growing slightly better than those expressing other genes, possibly because they are typical E2Fs with transcriptional activation domains that bind to downstream target genes (**Figure 5**). The results of LUC assay of E2F binding to the downstream *MSHs* gene and the phenotype of each *msh* mutant seedling under cadmium stress further indicate that E2F responds to cadmium stress in yeast by mobilizing its downstream MSH components.

Cadmium disturbs epigenetic modification and induces DNA damage in mouse preimplantation embryos and soybeans (Zhao et al., 2020; Cheng et al., 2021; Zhu et al., 2021). Cadmium exposure can affect oxidative stress, cell cycle arrest, DNA damage, and apoptosis in green crabs (*Scylla paramamosain*). Once cadmium-induced DNA damage is detected in plants, DDR is triggered. DDR is a cellular response to DNA damage, including regulation of DNA damage recognition and recruitment of DNA repair factors. Inhibition of cell cycle regulatory genes during DDR contributes to cell proliferation arrest (Jackson and Bartek, 2009; Schade et al., 2019). Therefore, genes related to mismatch repair and cell cycle regulation are, in principle, located in the nucleus. Our experiments demonstrated that *OsMSH2/3/6/7* are localized in the nucleus and function together with cell cycle regulatory genes in response to cadmium stress (**Figure 6**).

**Figure 7:**
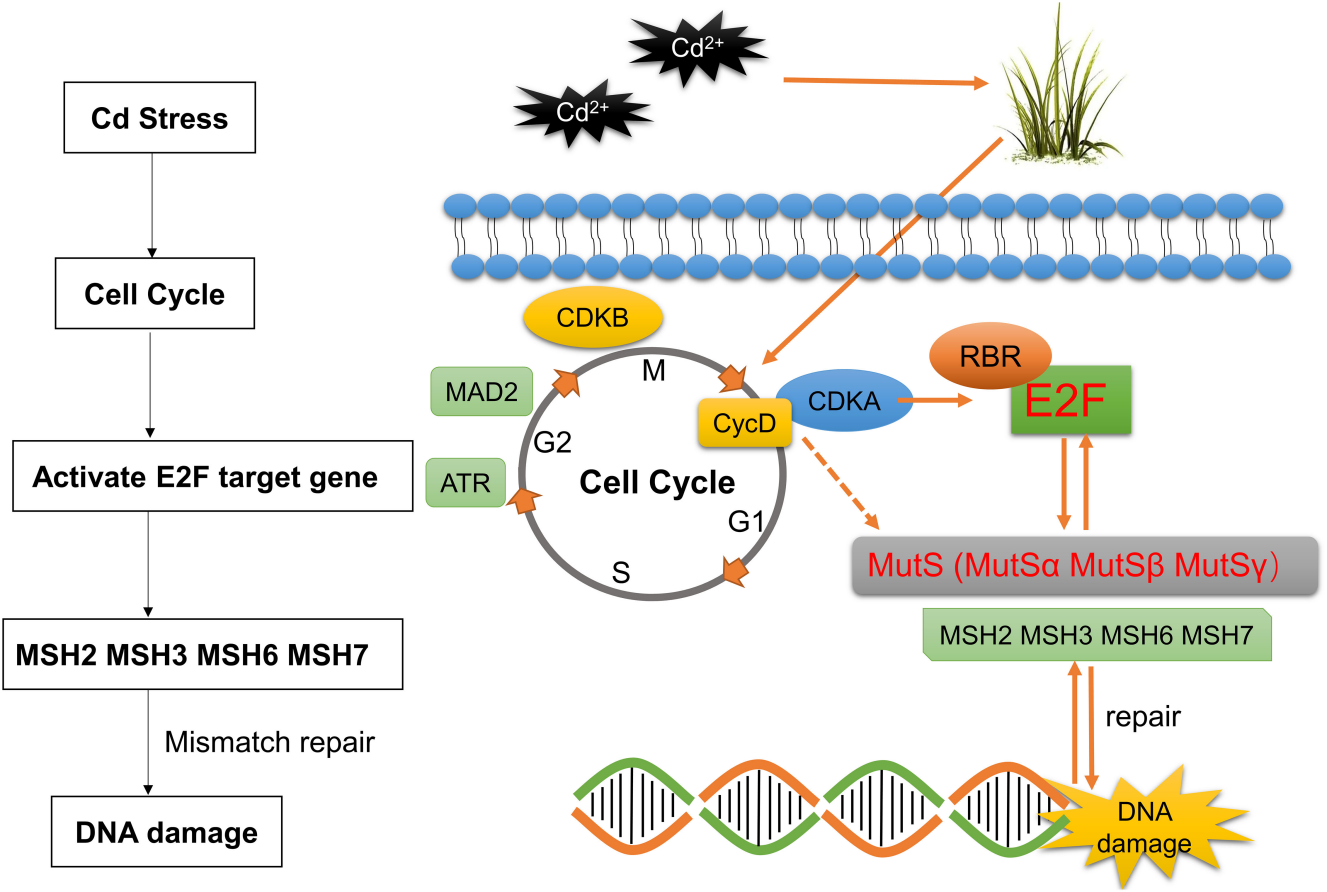
Model of Cd-induced cell cycle regulation in rice leaves. The solid line represents the critical pathway, the dashed line represents the possible pathway, and the technical route to the model is explained on the left.

Adaptation to changes in cellular nutrition and external environments is a fundamental cellular property. This adaptation requires the coordination of multiple networks among metabolic, growth, and cell cycle regulators, including the CDKfamily, members of the RBfamily, and E2F transcription factors (Huber et al., 2021). In response to external growth stimuli, including plant hormones, the abundance of specific G1 cell cycle proteins increases (Riou-Khamlichi et al., 2000). The CYCD-CDKA;1 complex phosphorylates RBR at multiple conserved sites, releasing activator E2F from bound RBR, thereby inducing the expression of downstream cell cycle genes (Nakagami et al., 2002; Magyar et al., 2012). Our qRT-PCR further confirmed the RNA-seq results, showing significant upregulation of *CDKA;1* and *CYCD* as the front end of G1/S activation, significant downregulation of *RBR* as the response to being phosphorylated, and significant upregulation of *OsE2Fa-1* and *OsE2Fa-2* due to being released from RBR (**Figures S2B, C**). All the results of LUC assay, yeast spot plate assay, and qRT-PCR indicated that the mobilization of E2F transcription factors is the key factor responsible for significant upregulation of *OsMSH2*, *OsMSH3*, *OsMSH6*, or *OsMSH7* under cadmium stress. *OsMSH2*, *OsMSH3*, *OsMSH6*, or *OsMSH7* were significantly downregulated in rice leaves in response to cadmium stress, contrary to their expression patterns in *Arabidopsis* roots. This discrepancy is possibly because cadmium stress induces different damages in rice leaves and *Arabidopsis* roots or because rice and *Arabidopsis* have different regulation patterns in response to cadmium stress. As shown in **Figure S2A**, the rice MMR system was not damaged, and *MSH2/3/6/7* was upregulated to repair DNA damage. A fully functional MMR system can regulate the G2/M phase by upregulating G2/M regulatory proteins and/or by activating *p53*, *ATM*, and *ATR* signaling pathways in human cells in response to exogenous and endogenous stresses. Therefore, we selected key G2/M genes (*CDKB* and *MAD2*) and other DNA stress sensors that coordinate stress responses with cell cycle checkpoint *ATR* genes (Wang et al., 2013). We found that both *CDKB* and *MAD2* were significantly downregulated after the knockdown of either MSH genes (**Figures 4L-M**) and significantly upregulated under cadmium stress, suggesting that MSH genes are also involved in the cell cycle regulation of G2/M. *OsMSH2* is significantly downregulated in *Osmsh3*, *Osmsh6*, and *Osmsh7* mutant rice, indicating an important role of *OsMSH2* in MutSα, MutSβ, and MutSγ complexes. *OsMSH3*, *OsMSH6*, and *OsMSH7* expression levels are altered in all mutant rice, indicating that the MutS system is a comprehensive complex involving the whole body (**Figures 4B-E**). Interestingly, *CycD5;2* expression was significantly upregulated in wild-type rice under cadmium stress and significantly downregulated in all mutants. Moreover, their expression was further significantly downregulated in the leaves after exposure of the mutants to cadmium stress. The expression of *CYCD-CDKA* genes was also changed under cadmium stress, as shown in transcriptome and qRT-PCR analyses. Therefore, we speculate that *CYCD-CDKA* may directly target and regulate MSH genes in G1/S for DNA damage repair in addition to activating downstream RBR phosphorylation (**Figures 4B-E**). However, this speculation needs to be proven by additional tests at a later stage. Based on our findings, we propose that the *CDKA-RBR-E2F-MSH* pathway may be the primary mechanism for enhancing cadmium stress tolerance in rice by mobilizing cell cycle factors of downstream key mismatch repair genes (**Figure 7**).

In conclusion, we systematically categorized and analyzed rice E2F genes by bioinformatics and demonstrated that rice E2F transcription factor can activate MSHs target genes. The cadmium stress experiments in rice seedlings and the yeast heterologous expression system demonstrated that E2F transcription factors and MSHs can respond to cadmium stress and elucidated the specific link between E2F and MSHs from transcriptomic and quantitative perspectives. Furthermore, our study provided a preliminary theoretical basis for revealing the intrinsic mechanism of E2F transcription factors responding to cadmium stress by binding to MSH mismatch repair genes E2F in the cell cycle. Next, we will take this as a breakthrough to further explore the relationship between E2F factor and cadmium stress in rice, and study the relationship between E2F and MSH in rice and other crops from multiple perspectives and layers. Our work paved a novel way for expanding the theoretical basis of the plant cell cycle and provided a theoretical reference for the study of the mechanism responding to cadmium stress in rice.

## Data availability statement

The datasets presented in this study can be found in online repositories. The names of the repository/repositories and accession number(s) can be found below: NCBI Sequence Read Archive under the BioProject identification number PRJNA890021.

## Author contributions

B-GM and B-RZ designed the experiments. W-JZ carried out most of the experiments. W-QL, YP, YS, LT, C-TL, DZ, L-JZ and J-HL. assisted in phenotypic identification and protein interaction tests. W-QL and Z-CY assisted in field management. W-JZ wrote the manuscript. B-GM and B-RZ revised and approved the final version of the manuscript. All authors contributed to the article and approved the submitted version.

## Funding

This work was supported by National Natural Science Foundation of China (32172042), Natural Science Foundation of Hunan Province (2021JJ30487), the earmarked fund for China Agriculture Research System. The authors would like to thank TopEdit (www.topeditsci.com) for its linguistic assistance during the preparation of this manuscript.

## Conflict of interest

The authors declare that the research was conducted in the absence of any commercial or financial relationships that could be construed as a potential conflict of interest.

## Publisher’s note

All claims expressed in this article are solely those of the authors and do not necessarily represent those of their affiliated organizations, or those of the publisher, the editors and the reviewers. Any product that may be evaluated in this article, or claim that may be made by its manufacturer, is not guaranteed or endorsed by the publisher.

## Supplementary material

Supplementary Figure 1 Amino acid sequence analysis of rice and *Arabidopsis* E2Fs. According to amino acid similarity, gray, green and orange represent identical or similar amino acids. Highly homologous DNA-binding structural domains that are repeated twice in rice and *Arabidopsis* E2F family proteins, as well as conserved regions, are marked.

Supplementary Figure 2 Expression profiles of cell cycle-related genes in response to Cd stress in wild-type leaves of rice and statistics of rice root length differences after Cd stress. **(A–C)** Statistics of differential expression of cell cycle-related genes in rice wild-type leaves under 2 mg and 4 mg Cd stress. Data were shown mean ± SD at least three independent experiments, and house-keeping gene Osactin was used as an internal control. * and ^#^ significantly statistical difference from the WT control and the corresponding mutant control, respectively (P < 0.05). **(D)** Statistics of root length differences between rice msh mutant and wild-type seedlings under 2 mg and 4 mg stress. (Student’s t-test, p < 0.05).

Supplementary Figure 3 Statistical analysis of the number of differenced genes in rice wild-type and MSH mutants under 2mg and 4mg cadmium stress **(A)** Overall statistics of the number of up and down-regulated genes **(B)** Venn diagram statistics of differential gene intersections and non-intersections between specific wild types and individual mutants
