# Supplementary material for: E2Fs co-participate in cadmium stress response through activation of MSHs during the cell cycle
Source: Front Plant Sci. 2022 Nov 29;13:1068769. doi: 10.3389/fpls.2022.1068769 (PMC9749859; doi:10.3389/fpls.2022.1068769)
Supplement: Supplementary file 1 [file Table_1.docx]

Table S1 List of primers

| Primer name | Primer Sequences (5’-3’) | Remark |
| --- | --- | --- |
| qMSH2-F2 | CGCAGGATTCGAGGGCTATT | qRT-PCR Primer of OsMSH2 |
| qMSH2-R2 | CTAACAAAAGGTTGCGGGCG |  |
| qMSH3-F2 | ATCCTCTGCCACGGAAAAGC | qRT-PCR Primer of OsMSH3 |
| qMSH3-R2 | GTTGGAGCATCAACCCCAGA |  |
| qMSH6-F2 | AGGAGAAAAGTCGATGCGGG | qRT-PCR Primer of OsMSH6 |
| qMSH6-R2 | AACTTTTCTGCTTGCCGCTG |  |
| qMSH7-F2 | GGCTGGCAGACCCACTTTAT | qRT-PCR Primer of OsMSH7 |
| qMSH7-R2 | GCCAATCTCAGCATCCACCT |  |
| qCycD5; 2F1 | GCTGCTCCTCTTCTTCCTCC | qRT-PCR Primer of CycD5; 2 |
| qCycD5; 2R1 | AAGTAGGCGATGGCGAGGTA |  |
| qCDKA;1F1 | CAGCATCGCAACATCGTCAG | qRT-PCR Primer of CDKA;1 |
| qCDKA;1R1 | GCGGTGGTTCTTGAAATCCG |  |
| qOsRBR1F1 | ATGTCATCCTTGGGCAGTGG | qRT-PCR Primer of OsRBR1 |
| qOsRBR1R1 | GCAGAACTGTGGCATCTCCT |  |
| qATRF1 | TCTCTCGCTTCTTCGCACAG | qRT-PCR Primer of ATR |
| qATRR1 | GAAGGATCCGCAGAGGCATT |  |
| qCDKB2;1F2 | CCTCAAGAAGTTCATCCGCG | qRT-PCR Primer of CDKB2;1 |
| qCDKB2;1R2 | GTTATGCGGCTTGAGGTCAC |  |
| qE2Fa-1F1 | ATATCGAGGAGGGGCTGGTC | qRT-PCR Primer of E2Fa-1 |
| qE2Fa-1R1 | AGGCCCAGCTTTGTTGTTCT |  |
| qE2Fa-2F2 | TTGTGCGCTAGTTCTCCAGG | qRT-PCR Primer of E2Fa-2 |
| qE2Fa-2R2 | AATCAACTATGCCGCCAGGT |  |
| qMAD2F2 | AGGTGAAGAAGTACGGCCTC | qRT-PCR Primer of MAD2 |
| qMAD2R2 | CCTTGCTCATGATCACCAGC |  |
| LOC-MSH2-BamHIF | gctctagaactagtggatccATGGAGGGCGACGACTTCTTGC | Primers for the construction of OsMSH2 subcellular localization vector |
| LOC-MSH2-Sa1IR | ggccccccctcgaggtcgacTCAAAGGAACTGCTGAAGCCAA |  |
| LOC-MSH3-BamHIF | gctctagaactagtggatccatgggcaagccgaagcagcaag | Primers for the construction of OsMSH3 subcellular localization vector |
| LOC-MSH3-Sa1IR | ggccccccctcgaggtcgacACATAAACTAAATGCTTCACAT |  |
| LOC-MSH6-BamHIF | gctctagaactagtggatccatggcgtcgccgtcgtcgcgc | Primers for the construction of OsMSH6 subcellular localization vector |
| LOC-MSH6-Sa1IR | ggccccccctcgaggtcgacttctccaatgacctgcacctta |  |
| LOC-MSH7-BamHIF | gctctagaactagtggatccATGCAGCCGCGGCGGCGGCA | Primers for the construction of OsMSH7 subcellular localization vector |
| LOC-MSH7-SaI1R | ggccccccctcgaggtcgacCCTCCGTTTCCTGAAGTGAG |  |
| MSH2-gRNA F1 | GGCAGAGAGGACAGACCGTACAT | Primers for the construction of the Crispr-Ca9 vector on MSH2 |
| MSH2-gRNA R1 | AAACATGTACGGTCTGTCCTCTC |  |
| MSH2-gRNA F2 | GCCGGAGGGCGACGACTTCTTGC |  |
| MSH2-gRNA R2 | AAACGCAAGAAGTCGTCGCCCTC |  |
| MSH3-gRNA F1 | GGCAGCATCCCTACCTTCCGCTT | Primers for the construction of the Crispr-Ca9 vector on MSH3 |
| MSH3-gRNA R1 | AAACAAGCGGAAGGTAGGGATGC |  |
| MSH3-gRNA F2 | GCCGATGCTCCAACTGTTGAGAA |  |
| MSH3-gRNA R2 | AAACTTCTCAACAGTTGGAGCAT |  |
| MSH6-gRNA F1 | GCCGTGGTACGAGGGGAGGGTGG | Primers for the construction of the Crispr-Ca9 vector on MSH6 |
| MSH6-gRNA R1 | AAACCCACCCTCCCCTCGTACCA |  |
| MSH6-gRNA F2 | GGCATTGAGTGAGCTAATTGAAG |  |
| MSH6-gRNA R2 | AAACTTCAATTAGCTCACTCAA |  |
| MSH7-gRNA F1 | GGCAGGAGCCCCTGAAGTCCTTA | Primers for the construction of the Crispr-Ca9 vector on MSH7 |
| MSH7-gRNA R1 | AAACTAAGGACTTCAGGGGCTCC |  |
| MSH7-gRNA F2 | GCCGCCATGTTGATTGCAGAAGA |  |
| MSH7-gRNA R2 | AAACTCTTCTGCAATCAACATGG |  |
| 62SK-OsE2Fa-1-BamHIF | TCTAGAACTAGTGGATCCATGGCGGGGAGTGGGAGG | Primers for LUC vector construction on OsE2Fa-1 |
| 62SK-OsE2Fa-1-EcoRIR | AAGCTTGATATCGAATTCCTAGGGTTTATGCATGTCTG |  |
| 62SK-OsE2Fa-2-BamHIF | TCTAGAACTAGTGGATCCATGGCGGCCGCCGGCGC | Primers for LUC vector construction on OsE2Fa-2 |
| 62SK-OsE2Fa-2-EcoRIR | AAGCTTGATATCGAATTCTCAGCTTATGCAGGAGTCTCC |  |
| 62SK-OsE2Fa-3-BamHIF | TCTAGAACTAGTGGATCCATGTCGTCGGGAGGAGGCCGT | Primers for LUC vector construction on OsE2Fa-3 |
| 62SK-OsE2Fa-3-EcoRIR | AAGCTTGATATCGAATTCTTATCCATGGTTGAAGCCCAC |  |
| 62SK-OsE2Fc-BamHIF | TCTAGAACTAGTGGATCCATGGACGGATCCGCCGCCAT | Primers for LUC vector construction on OsE2Fc |
| 62SK-OsE2Fc-EcoRIR | AAGCTTGATATCGAATTCTTAAGTGCCCCATGTATCAG |  |
| 62SK-OsE2Fe-1-BamHIF | TCTAGAACTAGTGGATCCATGGCGACGGCGGCGGTGAT | Primers for LUC vector construction on OsE2Fe-1 |
| 62SK-OsE2Fe-1-EcoRIR | AAGCTTGATATCGAATTCCTACAAAAACTGGTTAATGAC |  |
| 62SK-OsE2Fe-2-BamHIF | TCTAGAACTAGTGGATCCATGGCCGCCGCCGCCGAT | Primers for LUC vector construction on OsE2Fe-2 |
| 62SK-OsE2Fe-2-EcoRIR | AAGCTTGATATCGAATTCTTATGATGCTCTGTCTCGCCT |  |
| 0800-pOsMSH-HindIIIF | gacggtatcgataagcttACGTCCCCCCGTTTCCCG | Primers for LUC vector construction on pOsMSH-LUC |
| 0800-pOsMSH-BamHIR | tctagaactagtggatccGGCGGGGTGGGGCGGGAAAAG |  |
| pYES2-E2Fa-1-KpnI-F | TATTAAGCTTGGTACCATGGCGGGGAGTGGGAGGCCG | Primers for the construction of a yeast expression vector for OsE2Fa-1 |
| pYES2-E2Fa-1-BamHI-R | CGGCCGTTACTAGTGGATCCCTAGGGTTTATGCATGTCTGAG |  |
| pYES2-E2Fa-2-KpnI-F | AATATTAAGCTTGGTACCATGGCGGCCGCCGGCGCCGGCT | Primers for the construction of a yeast expression vector for OsE2Fa-2 |
| pYES2-E2Fa-2-BamHI-R | CGGCCGTTACTAGTGGATCCTCAGCTTATGCAGGAGTCTCCA |  |
| pYES2-E2Fa-3-KpnI-F | TATTAAGCTTGGTACCATGTCGTCGGGAGGAGGCCGTC | Primers for the construction of a yeast expression vector for OsE2Fa-3 |
| pYES2-E2Fa-3-BamHI-R | CGGCCGTTACTAGTGGATCCTTATCCATGGTTGAAGCCCAC |  |
| pYES2-E2Fc-KpnI-F | TATTAAGCTTGGTACCATGGACGGATCCGCCGCCATCC | Primers for the construction of a yeast expression vector for OsE2Fc |
| pYES2-E2Fc-BamHI-R | CGGCCGTTACTAGTGGATCCTTAAGTGCCCCATGTATCAGTC |  |
| pYES2-E2Fe-1-KpnI-F | TATTAAGCTTGGTACCATGGCGACGGCGGCGGTGATGG | Primers for the construction of a yeast expression vector for OsE2Fe-1 |
| pYES2-E2Fe-1-BamHI-R | CGGCCGTTACTAGTGGATCCCTACAAAAACTGGTTAATGACA |  |
| pYES2-E2Fe-2-KpnI-F | ATATTAAGCTTGGTACCATGGCCGCCGCCGCCGATGCTC | Primers for the construction of a yeast expression vector for OsE2Fe-2 |
| pYES2-E2Fe-2-BamHI-R | CGGCCGTTACTAGTGGATCCTTATGATGCTCTGTCTCGCCTG |  |
