# Supplementary material for: E2Fs co-participate in cadmium stress response through activation of MSHs during the cell cycle
Source: Front Plant Sci. 2022 Nov 29;13:1068769. doi: 10.3389/fpls.2022.1068769 (PMC9749859; doi:10.3389/fpls.2022.1068769)
Supplement: Supplementary file 2 [file Table_2.docx]

Table S2 The accession number of the gene.

| Gene name | Login ID |
| --- | --- |
| *OsMSH2* | LOC_Os05g19270 |
| *OsMSH3* | LOC_Os04g58630 |
| *OsMSH6* | LOC_Os09g24220 |
| *OsMSH7* | LOC_Os01g08540 |
| *OsE2Fa-1* | LOC_Os02g33430 |
| *OsE2Fa-2* | LOC_Os12g06200 |
| *OsE2Fa-3* | LOC_Os04g33950 |
| *OsE2Fc* | LOC_Os04g02140 |
| *OsE2Fe-1* | LOC_Os02g50630 |
| *OsE2Fe-2* | LOC_Os06g13670 |
