# Supplementary material for: E2Fs co-participate in cadmium stress response through activation of MSHs during the cell cycle
Source: Front Plant Sci. 2022 Nov 29;13:1068769. doi: 10.3389/fpls.2022.1068769 (PMC9749859; doi:10.3389/fpls.2022.1068769)
Supplement: Supplementary file 3 [file Table_3.docx]

Table S3 Effects of Cd treatment on seedling germination, fresh weight, dry weight and plant growth of rice.

| Lines of Oryza sativa | Germination percentage/% | Cd concentration/mg·L^-1^ | Plant height /cm | Root length/cm | Fresh weight of plantlet /mg | Dry weight of plantlet /mg |
| --- | --- | --- | --- | --- | --- | --- |
| WT | 90.63±2.08 | 0 | 31.66±0.61 | 10.66±0.36 | 0.324±0.016 | 0.055±0.003 |
|  |  | 2 | 28.12±1.22** | 9.94±016** | 0.270±0.020** | 0.048±0.003** |
|  |  | 4 | 26.46±1.33** | 9.36±0.26** | 0.266±0.024** | 0.044±0.005** |
|  |  |  |  |  |  |  |
| *Osmsh2* | 88.75±2.29 | 0 | 29.78±0.47 | 10.86±0.64 | 0.306±0.016 | 0.042±0.003 |
|  |  | 2 | 25.69±0.72** | 9.94±0.56* | 0.246±0.034** | 0.040±0.002* |
|  |  | 4 | 23.41±1.06** | 9.48±0.62** | 0.238±0.032** | 0.038±0.003** |
|  |  |  |  |  |  |  |
| *Osmsh3* | 89.58±2.08 | 0 | 29.01±0.99 | 10.32±0.18 | 0.294±0.044 | 0.045±0.004 |
|  |  | 2 | 25.03±1.09** | 9.38±0.38** | 0.238±0.022** | 0.035±0.003** |
|  |  | 4 | 23.30±0.86** | 8.94±0.36** | 0.228±0.032** | 0.033±0.002** |
|  |  |  |  |  |  |  |
| *Osmsh6* | 87.92±2.50 | 0 | 33.37±0.58 | 9.64±0.36 | 0.298±0.026 | 0.043±0.002 |
|  |  | 2 | 29.27±1.08** | 8.74±0.46** | 0.270±0.020* | 0.036±0.002** |
|  |  | 4 | 27.29±1.36** | 8.30±0.50** | 0.244±0.036** | 0.035±0.003** |
|  |  |  |  |  |  |  |
| *Osmsh7* | 90.42±2.92 | 0 | 32.49±0.46 | 9.92±0.32 | 0.322±0.018 | 0.052±0.002 |
|  |  | 2 | 28.41±1.00** | 9.04±0.56** | 0.254±0.036** | 0.038±0.002** |
|  |  | 4 | 26.48±0.65** | 8.52±0.58** | 0.252±0.018** | 0.037±0.003** |
