# Supplementary figures and images for: E2Fs co-participate in cadmium stress response through activation of MSHs during the cell cycle
Source: Front Plant Sci. 2022 Nov 29;13:1068769. doi: 10.3389/fpls.2022.1068769 (PMC9749859; doi:10.3389/fpls.2022.1068769)

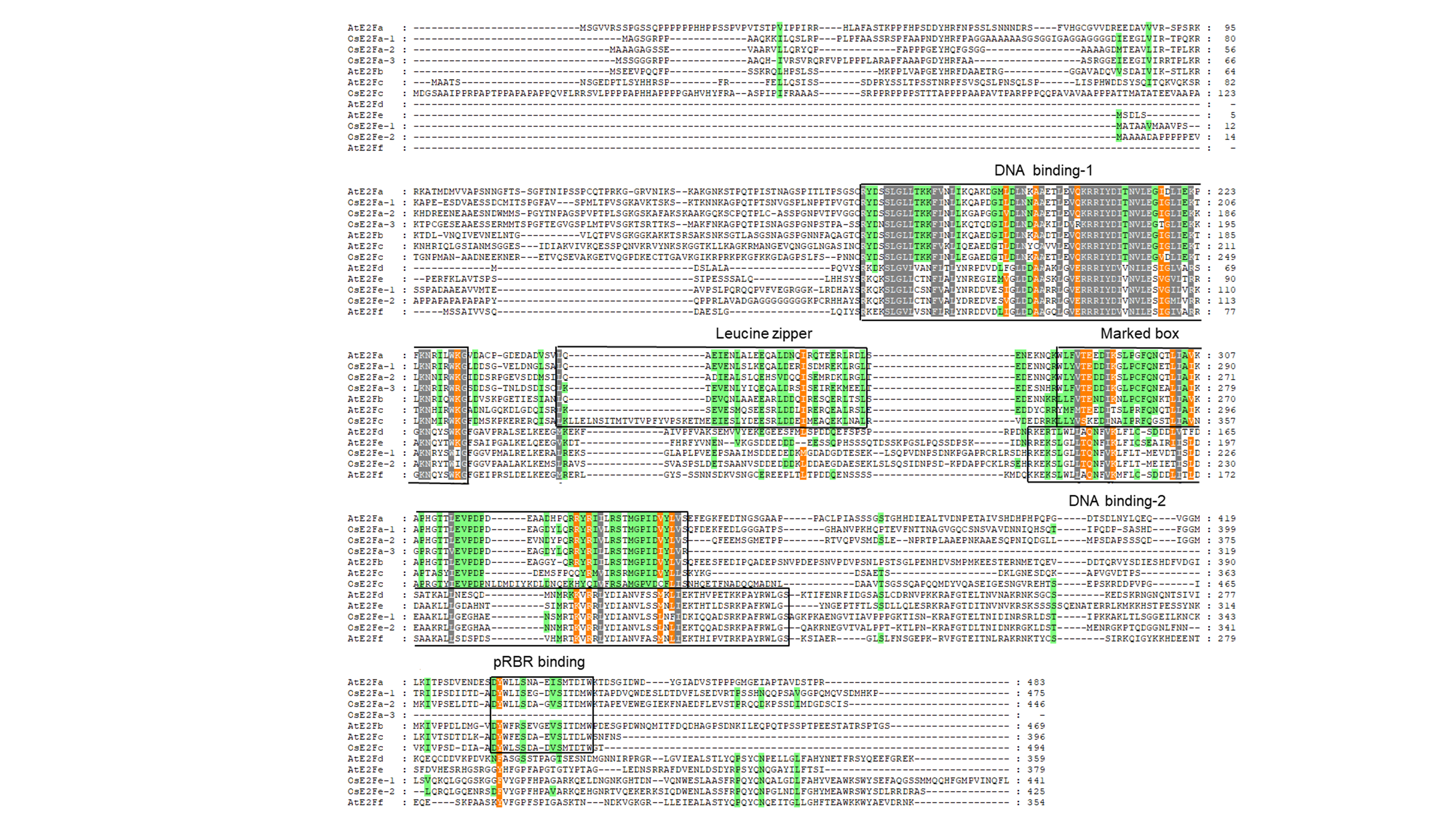

Supplement: Supplementary Figure 1 — Amino acid sequence analysis of rice and Arabidopsis E2Fs. According to amino acid similarity, gray, green and orange represent identical or similar amino acids. Highly homologous DNA-binding structural domains that are repeated twice in rice and Arabidopsis E2F family proteins, as well as conserved regions, are marked. [file Image_1.tif]

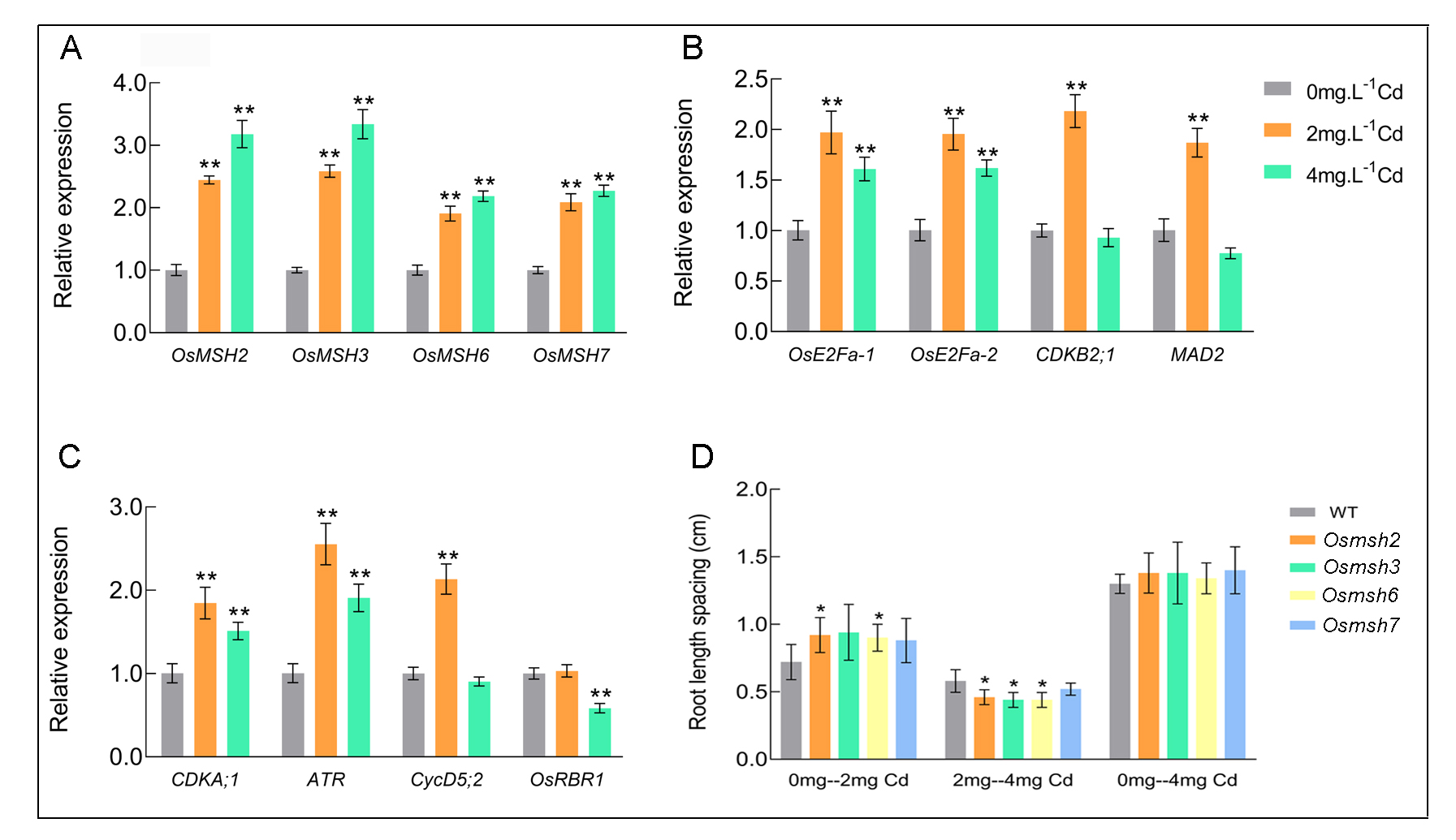

Supplement: Supplementary Figure 2 — Expression profiles of cell cycle-related genes in response to Cd stress in wild-type leaves of rice and statistics of rice root length differences after Cd stress. (A–C) Statistics of differential expression of cell cycle-related genes in rice wild-type leaves under 2 mg and 4 mg Cd stress. Data were shown mean ± SD at least three independent experiments, and house-keeping gene Osactin was used as an internal control. * and # significantly statistical difference from the WT control and the corresponding mutant control, respectively (P < 0.05). (D) Statistics of root length differences between rice msh mutant and wild-type seedlings under 2 mg and 4 mg stress. (Student’s t-test, p < 0.05). [file Image_2.jpeg]

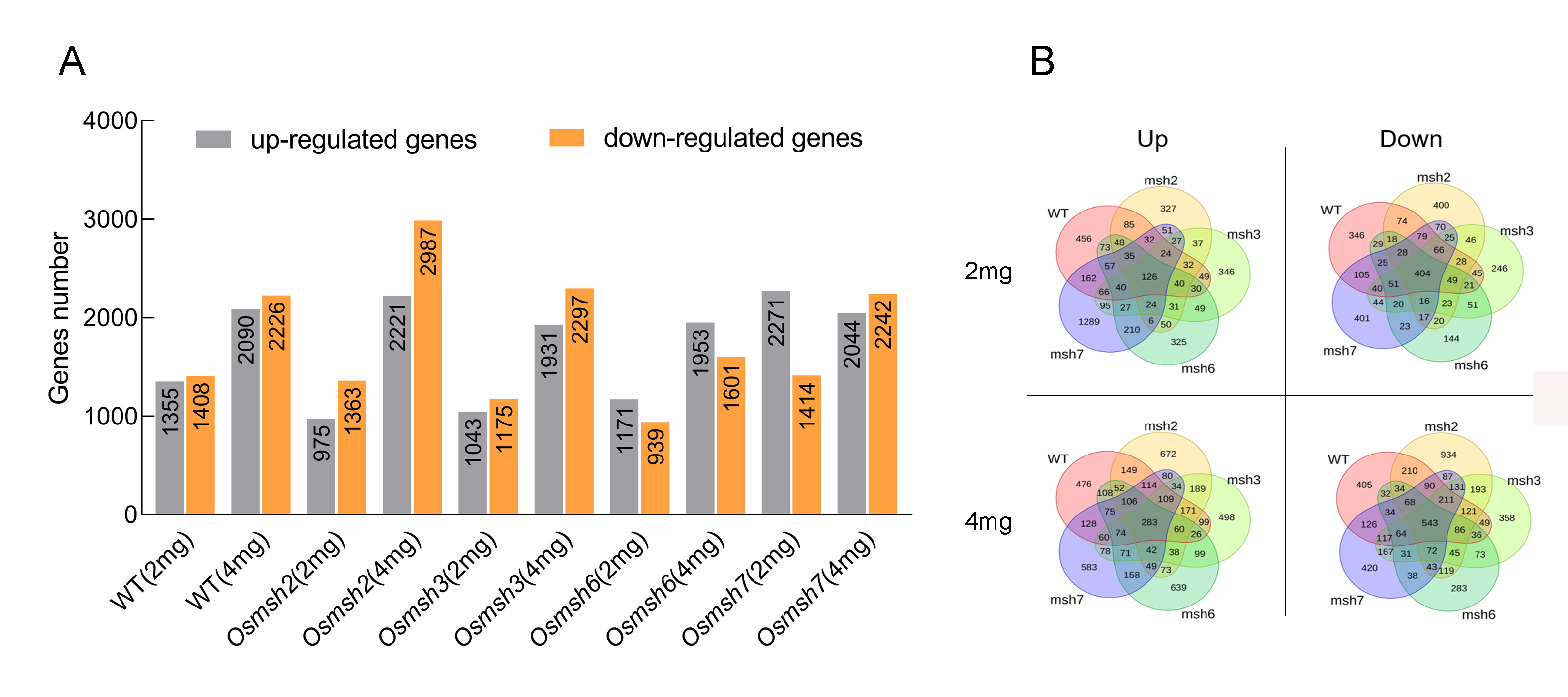

Supplement: Supplementary Figure 3 — Statistical analysis of the number of differenced genes in rice wild-type and MSH mutants under 2mg and 4mg cadmium stress (A) Overall statistics of the number of up and down-regulated genes (B) Venn diagram statistics of differential gene intersections and non-intersections between specific wild types and individual mutants [file Image_3.jpeg]
